# A novel variant in *BCL11B* in an individual with neurodevelopmental delay: A case report

**DOI:** 10.1002/mgg3.2132

**Published:** 2023-01-23

**Authors:** Yonglin Yu, Xiaoyi Jia, Hongwei Yin, Hongfang Jiang, Yu Du, Fan Yang, Zuozhen Yang, Haifeng Li

**Affiliations:** ^1^ Department of Rehabilitation, the Children's Hospital Zhejiang University School of Medicine, National Clinical Research Center for Child Health Hangzhou China; ^2^ Cipher Gene LLC Beijing China

**Keywords:** BCL11B, cerebral palsy, developmental delay, whole‐exome sequencing

## Abstract

**Background:**

B‐Cell CLL/Lymphoma 11B (BCL11B) is a C_2_H_2_ zinc finger transcription factor that has broad biological functions and is essential for the development of the immune system, neural system, cardiovascular system, dermis, and dentition. Variants of *BCL11B* have been found in patients with neurodevelopmental disorders and immunodeficiency.

**Materials and Methods:**

Whole‐exome sequencing (WES) and clinical examinations were performed to identify the etiology of our patient. A variant in the *BCL11B* gene, NM_138576.4: c.1206delG (p.Phe403Serfs*2) was found and led to frameshift truncation.

**Results:**

We reported a male patient with developmental delay and cerebral palsy who carried the *BCL11B* variant. The detailed clinical features, such as brain structure and immune detection, were described and reviewed in comparison to previous patients.

**Conclusions:**

The BCL11B‐related neurodevelopmental disorders are rare, and only 17 variants in 25 patients have been found to date. Our report expands the variants spectrum of *BCL11B* and increases the case of neurodevelopmental abnormalities.

## Graphical abstract

The BCL11B‐related neurodevelopmental disorder was rare, only 14 variants were found to date. Our report expands the variants spectrum of BCL11B and increases the case of neurodevelopmental abnormalities.

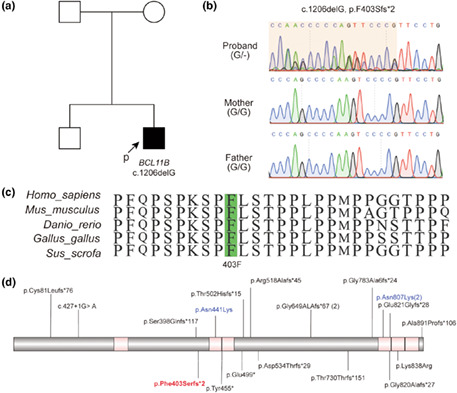

## 1 INTRODUCTION

BCL11B (B‐Cell CLL/Lymphoma 11B) encodes a C_2_H_2_‐type zinc finger protein; it is a homolog gene for BCL11A that was first identified in 2001 (Satterwhite et al., 2001). BCL11B is a zinc finger transcription factor, and its functions are most widely studied in the immune and nervous systems. Bcl11b is widely expressed in various subsets of T‐cell and plays a crucial role in the regulation of T‐cell development, function, and survival (Avram & Califano, 2014). In neuronal networks, Bcl11b has also been shown to be expressed in corticospinal motor neurons (CSMNs). Based on a study of Bcl11b‐deficient mice, Bcl11b is required to establish cortical connections to the spinal cord (Arlotta et al., 2005). In addition, conditional deletion of Bcl11b in the mouse hippocampus leads to the loss of excitatory synapses and abnormal synaptic transmission (De Bruyckere et al., 2018).

The first patient with the *BCL11B* variant, who presented with “leaky” SCID, was reported in 2016. Zebrafish functional experiments revealed that a *BCL11B* variant caused human multisystem anomalies with SCID and that BCL11B was crucial in hematopoietic progenitors (Punwani et al., 2016). Subsequently, successive reports demonstrated the pathogenicity of *BCL11B* variants (Alfei et al., 2021; Lessel et al., 2018; Lu et al., 2021; Prasad et al., 2020; Qiao et al., 2019; Yang et al., 2020). All patients presented with developmental delay, most of whom presented with intellectual disability, mild facial dysmorphisms, and impaired T‐cell development. Variants in *BCL11B* are now associated with “Immunodeficiency 49 (OMIM: 617237)” and “Intellectual developmental disorder with speech delay, dysmorphic facies, and T‐cell abnormalities (OMIM: 618092)”. However, the reported patients and variants are limited. Further expansion of variant sites and phenotypes is beneficial for a better understanding of this disease.

Here, we report a four‐year‐ and five‐month‐old boy who presented with cerebral palsy and global developmental delay and was identified with a de novo variant in *BCL11B*. The frameshift variant NM_138576.4: c.1206delG (p.Phe403Serfs*2) was predicted to cause protein truncation (p.Phe403Serfs*2) and possibly disrupt the protein function.

## 2 MATERIALS AND METHODS

### 2.1 Patient

Written informed consent was obtained from the legal guardians of the patient to participate in this study. The study was approved by the Human Ethics Committees of the Children's Hospital affiliated with Zhejiang University. The clinical course, cerebral magnetic resonance imaging (MRI), and genetic testing of our patient were analyzed. The immunophenotype of our patient was determined by peripheral blood and serum.

### 2.2 Whole‐exome sequencing

Genetic analysis was performed using WES. The source of the sample was the peripheral blood of the patient and his parents. The genomic DNA library was captured using the IDT XGen Exome Research Panel. WES was then performed on the NovaSeq 6000 Sequencing platform using paired‐end reads and compared to the human reference genome (GRCh38/hg38). The variants were annotated by ANNOVAR (McKenna et al., 2010). The pathogenicity of the identified variants was predicted by multiple software programs, such as Mutation Taster, Polyphen2, and Sorting Intolerant from Tolerant (SIFT). All variants were filtered by inherent patterns, frequency, clinical characteristics, and public databases. Pathogenic variants were finally screened according to the American College of Medical Genetics and Genomics (ACMG) (Richards et al., 2015). Sanger sequencing was performed in the trio family. Sanger sequencing was performed to verify the variants we detected in WES.

## 3 RESULTS

### 3.1 Case description

**FIGURE 1:**
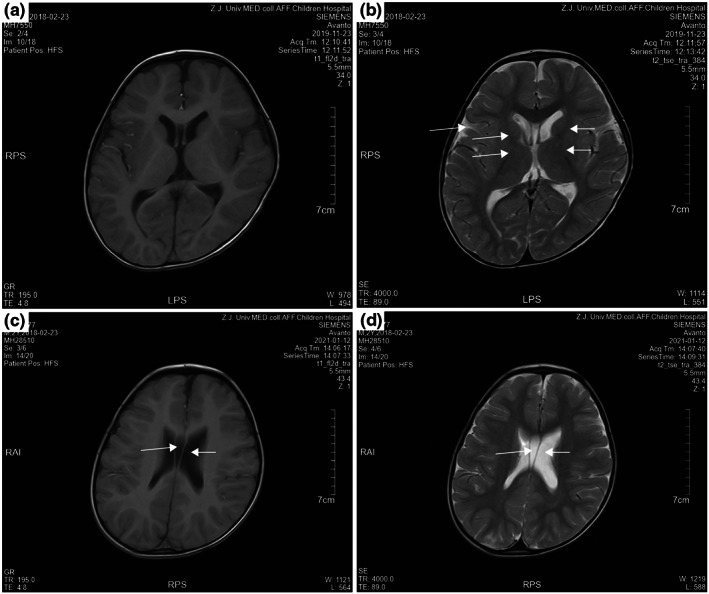
MRI results in our patient in 2019 (a, b) and 2021 (c, d). (a) T1‐weighted axial image. (b) T2‐weighted axial images. The widened extracerebral space and the internal capsule showed less signal reduction. (c, d) T1‐ and T2‐weighted axial images showed a widened extracerebral space and lucid septum cysts.

A 4‐year‐ and 5‐month‐old boy, with an older brother and nonconsanguineous parents in good health, initially presented with a developmental delay in motor and language when he was 9 months old. He could grab, but not pinch. He was unable to sit without support and unable to crawl, and he had an abnormal posture of standing on his tiptoes. Gesell Developmental Scale results (2019.4.25) showed cognitive and motor delays. MRI results (2019.11.23) showed widened extracerebral space and less signal reduction in the internal capsule (Figure 1a,b). The patient was diagnosed with mixed cerebral palsy and was followed by rehabilitation (2020.12.25‐2021.1.15; 2021.3.1‐2021.3.9). He made some cognitive and motor progress. A repeat MRI (2021.01.12) showed widened extracerebral space and a lucid septum cyst (Figure 1c,d). Immune detection (2021.7.21) showed high CD8 and low NK cells and IgG2 (Table 1). However, the patient had no obvious immunodeficiency phenotype. He had not experienced infections or allergies. After various rehabilitation therapy, such as sling exercise training, physiotherapy, occupational, and speech therapy, his development improved markedly. When he was 3 years and 3 months old, he could climb stairs, stand, and walk with support, and he could grasp objects with both hands, but with poor accuracy and coordination. He could name a few animals, and his vocabulary was more than 20 words, but he could not speak phrases. He had poor separation of the lower extremities. Currently, he has poor overall development, with the language area slightly stronger than the other areas, and the motor area slightly behind.

**TABLE 1:** The immune results in our patient (2021.7.21)

Cluster of differentiation	Value	Reference
CD19	24.4	18.5% ~ 28.0%
CD3	67.3	56.0% ~ 68.0%
CD4	29.7	29.0% ~ 40.0%
CD8	27.2↑	19.0% ~ 25.0%
CD3−CD16+CD56+	4.7↓	9.0% ~ 19.0%
CD4/CD8−	1.09↓	1.1–2.0
Immunoglobulin subclass		
IgG1	5.74	4.90 ~ 11.4 g/L
IgG2	1.16↓	1.50 ~ 6.40 g/L
IgG3	0.08↓	0.20 ~ 1.10 g/L
IgG4	0.80	0.08 ~ 1.40 g/L

### 3.2 Identification of gene variation

**FIGURE 2:**
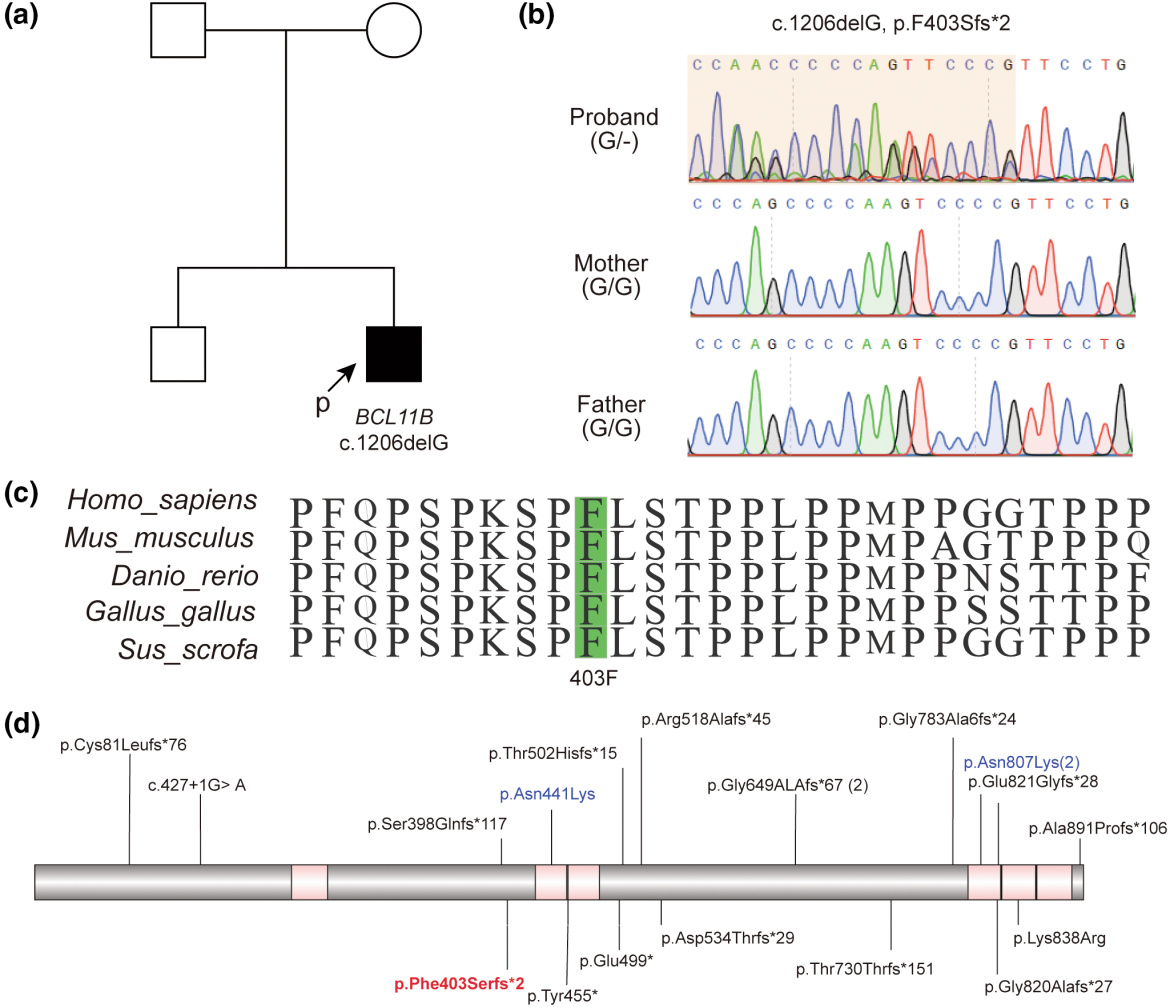
Identification of the variant in *BCL11B*. (a) The pedigree of our patient. The proband is highlighted with a filled square with an arrow. (b) Sanger sequencing in the trio family. A de novo 1206C deletion was identified by sequencing. (c) The changed 403F amino acid was conserved in multiple species. (d) Schematic of variants in the *BCL11B* gene in neurodevelopmental disease patients. The variant in our patient is shown in red text. Missense variants in the *BCL11B* gene are shown in blue text.

WES was performed to further identify the etiology of our patient. A heterozygous *BCL11B* variant in exon 4, NM_138576.4: c.1206delG (p.Phe403Serfs*2) was identified, resulting in a frameshift variation p.Phe403Serfs*2, which could be expected to result in protein truncation (Figure 2). The de novo variant in our patient was verified by the Sanger sequencing and his parents did not carry the variant. This variant was not reported or included in the gnomAD, ExAC, or ClinVar databases, indicating its rarity. The probability of being loss‐of‐function intolerant (pLI) in BCL11B was 0.99 and was considered an extremely intolerant transcript. Finally, the *BCL11B* variant in our patient was classified as likely pathogenic (PVS1_Strong+PS2_Moderate+PM2_Supporting) according to ACMG guidelines.

## 4 DISCUSSION

BCL11B is a zinc finger protein transcription factor with multiple functions in the development of the immune and nervous cutaneous systems. BCL11B plays an important role in the development of deep‐layer corticospinal projection neurons and striatal middle spiny neurons (Nikouei et al., 2016). Additionally, in mice, Bcl11b is highly expressed in medium spiny neurons (MSNs) and the hippocampus, in which Bcl11b (KO/KO) mouse MSNs fail to differentiate (Arlotta et al., 2008), and selective loss of Bcl11b expression in the adult hippocampus results in impaired spatial working memory (Simon et al., 2016). In addition, Bcl11b (KO/KO) mouse exhibit a defect in embryonic tooth development (Katsuragi et al., 2013).

Recent studies in *BCL11B* variant patients have presented an immunophenotype, a developmental delay, and other clinical features, such as abnormal facial appearance and dental anomalies, which are reviewed in Table 2. A total of 10 studies have reported 17 *BCL11B* variants and 25 patients, including 3 missense variants, 1 splice variant, and 13 truncated variants. These findings show an association between *BCL11B* variants and “Immunodeficiency 49” and “Intellectual developmental disorder with dysmorphic facies, speech delay, and T‐cell abnormalities.” Interestingly, patients with missense variants tend to have a more severe immunodeficiency, which may be due to the loss of DNA binding (Lessel et al., 2018). Our patient with *BCL11B* frameshift variants had no history of allergies or infections. A hematological evaluation of his immune system was performed (Table 1) and found some unusual changes were found. However, no immunodeficiency was found in our patient, which was also the same as previously reported cases (Lessel et al., 2018).

**TABLE 2:** *BCL11B* variants (NM_138576.4) and associated phenotypes in individuals with developmental delay

*BCL11B* variants	Ref.(PMID)	Age (year)	Gender	Autistic features	Speech impairment	Motor delay	Intellectual disability	Abnormal facial appearance	Refractive error	Dental anomalies	Immune response	MRI abnormal
p.N441KAsn441Lys	27959755	0	M	NA	+	+	+	+	NA	NA	+	Callosal agenesis
p.Gly820Alafs*27	29985992	3 11/12	F	+	+	+	+	+	+	+	−	−
p.Gly649Alafs*67		15 1/12	M	−	+	+	+	+	−	+	+	−
p.Ala891Profs*106		1 2/3	M	+	+	+	+	+	+	−	+	−
p.Thr502Hisfs*15		1 5/6	F	−	+	+	+	+	−	−	−	−
p.N807KAsn807Lys		2 1/4	M	−	+	+	+	+	−	+	+	−
p.Cys81Leufs*76		17 7/12	M	−	+	+	+	+	−	−	−	−
p.D534TfsAsp534Thrfs*29		13	F	+	+	+	+	+	−	−	−	−
46,XY,t(4;14)(p15;q32.1)		11	M	+	+	+	+	+	−	−	−	−
46,XY,t(4;14)(q31.1;q32.2)		29	M	−	+	−	+	+	−	−	−	Moderate ectopia of amygdala
p.GluE499*		6 1/2	F	−	+	+	+	+	+	+	−	−
p.TyrY455*		9 11/12	M	−	+	+	+	+	+	−	−	Hypoplasia of the globus pallidus
p.ArgR518Alafs*45		9 3/4	M	−	+	+	+	+	−	−	+	−
p.Thr730Thrfs*151	31347296	5	F	NA	+	+	+	+	NA	NA	+	−
p.Ser398GlnQfs*117	33194885	1 5/12	F	NA	+	+	+	+	NA	NA	+	Abnormal myelination of the white matter
p.Gly649Alafs*67	32659295	4	F	NA	+	+	+	+	NA	+	NA	Dysgenesis of the corpus callosum and enlargement of the frontal horns of the lateral ventricles bilaterally
p.N807KAsn807Lys		9	F	NA	+	+	+	+	+	+	−	−
p.N441KAsn441Lys	34844266	0	M	NA	+	+	+	+	+	−	NA	Corpus callosum dysgenesis, mildly enlarged frontal horns of lateral ventricles
p.Cys826TyrY	34887873	14	F	NA	−	−	+	−	−	−	+	−
p.Gly783Ala6fs*24	36275064	2 1/12	M	−	+	+	NA	+	−	−	+	Widening of the extracerebral interval, abnormal signals of the cornu occipitale and a reduced right‐brain cerebral volume
p.Lys838Arg	36202297	5	F	−	+	+	NA	NA	NA	NA	NA	Frontotemporal substance reduction with dilated CSF spaces
c.427+1G>A	36176959	11	F	+	+	+	+	+	NA	+	+	−
		3 10/12	M	−	+	+	+	+	NA	+	+	−
		35	F	−	+	+	+	+	NA	−	−	NA
p.Glu821Glyfs*28		3 8/12	M	−	+	+	+	+	NA	+	+	−
p.PheF403Serfs*2	Our report	4 5/12	M	−	+	+	+	−	−	−	−	Widened extracerebral space and the internal capsule showed less signal reduction

Abbreviation: NA, not known available.

The clinical features of patients with *BCL11B* gene variants are heterogeneous, and recent studies have suggested that craniosynostosis may be associated with this gene variant(Gaillard et al., 2021). Further research is required to clarify the relationship between craniosynostosis and BCL11B variation. Additionally, whether the brain structure of patients is abnormal also varies. Only five of the 19 patients reported thus far had abnormal MRI findings (Table 2). Our patient showed abnormal MRI results, with a widened extracerebral space in 2019 and lateral ventricle enlargement in 2021 (Figure 1). In addition to extending the abnormal phenotype on MRI, our patient was the second to be diagnosed with cerebral palsy among *BCL11B* variant patients. He presented with poor upper extremity control, bilateral foot eversion, standing on tiptoe, and toe grip. A previous case diagnosis of choreoathetotic cerebral palsy manifested as an extension of the arms with rotation of the wrist, the extension of the neck, flexion of the spine, and continuous dyskinetic movements of the tongue (Prasad et al., 2020). Richer clinical features are presented due to the patient carrying a missense variant (p.N807K), such as abnormal facial appearance, dental anomalies, dysgenesis of the corpus callosum, and bilateral enlargement of the frontal horns of the lateral ventricles bilaterally.

## 5 CONCLUSIONS

In summary, variants in the *BCL11B* gene showed high phenotypic heterogeneity, but the mechanisms that affect neurodevelopment remain limited. Our report expands the spectrum of DNA variants of the *BCL11B* gene and neurodevelopmental abnormalities of BCL11B‐related disorders. The clinical phenotype and gene variation spectrum need to be expanded through additional cases, and more detailed functions need to be studied in the future.

## AUTHOR CONTRIBUTIONS

Haifeng Li and Yonglin Yu conceived and designed the experiments. Xiaoyi Jia and Hongwei Yin did the patient recruitment and clinical analysis. Fan Yang and Zuozhen Yang did the WES and molecular analysis. Yonglin Yu and Hongfang Jiang wrote the first draft of the manuscript. All authors reviewed and approved the final manuscript.

## CONFLICT OF INTEREST

The authors declare that they have no competing interests.

## ETHICAL COMPLIANCE

The parents of the proband were informed by the consultant in the Department of Pediatric Neurology about the purpose of the DNA analysis. The samples were investigated after obtaining informed consent from the parents. This work was approved by the Children's Hospital affiliated with Zhejiang University.
